# Cannabis-Induced Catatonia: A Case Series

**DOI:** 10.7759/cureus.8603

**Published:** 2020-06-13

**Authors:** Hema Mekala, Zamaar Malik, Judith Lone, Kaushal Shah, Muhammad Ishaq

**Affiliations:** 1 Psychiatry, Griffin Memorial Hospital, Norman, USA; 2 Psychiatry, Medical University of the Americas, Camps, KNA

**Keywords:** synthetic cannabis, k2, cannabis, catatonia, addiction, thc, schizophrenia, benzodiazepine, ect, psychiatry

## Abstract

Catatonia is a psychomotor condition characterized by physical presentations ranging from severe immobility to excessive psychomotor agitation with an array of accompanying emotional aspects. Though initially thought to be a subform of schizophrenia, it is now recognized to be associated with many different psychiatric, neurological, and medical diagnoses. The emergence of catatonia is becoming more prevalent with its changing pattern and extensive use of recreational and illegal drugs. With the legalization of marijuana, its use is on the rise leading to several mental health conditions, including catatonia. If left untreated, catatonia has a significant morbidity and mortality rate; hence, prompt evaluation and diagnosis are critical for the prevention of adverse events. Benzodiazepines (BZDs) and electroconvulsive therapy (ECT) have been found to be most effective and remained as the preferred treatment options. In this paper, we present the case of two patients who presented with catatonia after cannabis consumption and discuss their treatment course and management.

## Introduction

Catatonia is a common condition observed in acutely ill patients with mental health conditions. In the United States, approximately 90,000 people tend to suffer from catatonia each year, with a prevalence ranging between 7.6% to 38% [1]. Catatonia is mostly evidenced in patients with delirium, mood disorders, schizophrenia, post-traumatic stress disorder (PTSD), and dissociative states [1,2]. It is most often characterized by decreased motor activity, lack of responsiveness, and posturing. Furthermore, it is classified into three categories, namely retarded, malignant, and excited catatonia [3]. Catatonia may also occur due to metabolic, neoplastic, infectious conditions, withdrawal or intoxication of prescription medications, and recreational drug use [2,3]. It is considered a medical and psychiatric emergency leading to severe rigidity, autonomic nervous system instability, and altered mental status resulting in death if untreated [1,2]. Even though diagnosing catatonia is straightforward from the clinical presentation, the etiology is often overlooked. This case series places emphasis mainly on cannabis-induced catatonia when considering a differential diagnosis.

## Case presentation

Case 1

A 22-year-old Caucasian woman with no known past psychiatric history was transferred from another facility for depression, suicidal ideation, psychosis, and bizarre behavior. On admission, she was withdrawn with minimal verbalization, despondent, had a flat affect, and exhibited thought blocking throughout the evaluation. Physical examination and laboratory studies showed no additional findings except for anemia and positive urine drug screen for marijuana.

After a thorough clinical evaluation, catatonia was considered as the underlying cause for her presentation, and she was started on lorazepam 0.5 mg by mouth (PO) every morning (QAM), sertraline 50 mg PO QAM (by mouth every morning), and olanzapine 5 mg by mouth (PO) each night at bedtime (QHS). She was later discontinued on sertraline and instead started on duloxetine 30 mg PO QAM (by mouth every morning), which was gradually titrated up to 120 mg PO QAM (by mouth every morning). The lorazepam was also increased to 1 mg by mouth (PO) twice a day (BID). The new regimen resulted in a significant improvement in her mood symptoms and resolution of catatonia. After recovery, the patient reported that she had no previous history of psychiatric and medical conditions and was not on any medications prior to her admission. She admitted to smoking marijuana daily to cope with the "stress" in her life for the past few years. Hence, in the absence of any other etiology, it was concluded that the patient's catatonic presentation, depression, and psychosis were attributed to her cannabis use.

Case 2

A 33-year-old Caucasian woman with a past psychiatric history of schizophrenia and depression was brought to the hospital by her husband for the inability to take care of herself. He reported that the patient was having bizarre disorganized behavior and a worsening of her baseline auditory hallucinations. 

According to her family, the patient was admitted to another facility for schizophrenia and was discharged ten days ago. Her medication compliance was unknown. On admission, the patient was unable to sit or talk, mumbled sometimes, and had a flat affect. We continued her home medication, olanzapine 5 mg PO BID (by mouth twice a day), and started her on lorazepam 1 mg PO BID (by mouth twice a day) for possible catatonia. The patient improved gradually and was able to communicate as her catatonia resolved. Upon further evaluation, the patient reported that after discharge from another facility, she smoked marijuana resin daily and did not know the events that led to her admission. Her worsening of psychosis and the onset of catatonia were associated with resin use.

## Discussion

Though the presentation of catatonia is evident in the case scenarios discussed above, identifying possible etiologies and preventing its recurrence was clinically challenging as it could occur due to a wide variety of medical and psychiatric conditions. 

With increasing legalization and marijuana use worldwide, including in the United States, we are likely to see an increased prevalence of catatonia along with other psychiatric conditions [4,5]. Cannabis products can be consumed in a number of ways, and they all have different potencies. The potency of cannabis depends on the concentration (%) of Δ9 -tetrahydrocannabinol (THC), which is the primary psychoactive component of the cannabis plant [6]. Different products of cannabis such as "buds" (dried cannabis flowers), oil (butane honey oil, shatter, wax, crumble), and resin (hashish, bubble hash) are available, and they are consumed either through smoking or as edibles [7].

Although no precise pathophysiological mechanisms of catatonia have been identified, studies have reported that it occurs primarily due to decreased activity of gamma-aminobutyric acid type A (GABA_A_) receptor, dopamine at the dopamine type-2 (D2) receptor, and glutamate at N-methyl-D-aspartate (NMDA) receptor [8]. A study on mice conducted by Fairbairn et al. showed that oral THC resulted in cataleptic events due to the increased availability of arachidonic acid, possibly from the gut, with a subsequent increase in the level of prostaglandin E2 (PGE2) [9]. Consumption of synthetic cannabinoid, also known as "spice" or "K2," has also led to catatonic presentations. Synthetic cannabinoids have had increased popularity in recent years due to their ease of availability, low cost, and lack of detection by standard urine drug screens [10]. A case report by Smith et al. mentioned that a 17-year-old male with no previous psychiatric history presented with catatonic symptoms after smoking about 2 g to 3 g per day of K2, which was resolved with lorazepam administration and six electroconvulsive therapy (ECT) treatments [11]. However, both of our patients denied using synthetic cannabinoids. 

Benzodiazepines (BZDs), especially lorazepam, are found to be extremely beneficial in resolving symptoms of different catatonic presentations by increasing GABA and decreas­ing NMDA receptor activity and are generally used as the first line of treatment [12]. Even though it is rare, the dosing of BZDs up to 20 mg to 30 mg per day is required for improvement or resolution of catatonic symptoms [13]. In patients unresponsive to BZDs, ECT has shown to be efficacious. Along with these interventions, avoiding exacerbating factors such as recreational or illegal drug use and dopamine blocking agents may help relieve catatonia symptoms [12].

## Conclusions

Although there is limited evidence thus far from the literature regarding cannabis use as a potential cause of catatonia, physicians should consider it as a potential etiology, as it is now one of the most commonly abused substances. Catatonia presentation could be a traumatic experience for the patients and their families. Hence, it is crucial to educate them about the potential risks associated with marijuana consumption and counsel against its use. Above all, clinicians should be vigilant about the use of any recreational drugs by their patients to provide optimal care. 
